# *Fasciola gigantica*–Derived Excretory-Secretory Products Alter the Expression of mRNAs, miRNAs, lncRNAs, and circRNAs Involved in the Immune Response and Metabolism in Goat Peripheral Blood Mononuclear Cells

**DOI:** 10.3389/fimmu.2021.653755

**Published:** 2021-04-12

**Authors:** Sha-Sha Wang, Dan Chen, Jun-Jun He, Wen-Bin Zheng, Ai-Ling Tian, Guang-Hui Zhao, Hany M. Elsheikha, Xing-Quan Zhu

**Affiliations:** ^1^ State Key Laboratory of Veterinary Etiological Biology, Key Laboratory of Veterinary Parasitology of Gansu Province, Lanzhou Veterinary Research Institute, Chinese Academy of Agricultural Sciences, Lanzhou, China; ^2^ College of Veterinary Medicine, Northwest A&F University, Yangling, China; ^3^ School of Science, Fudan University, Shanghai, China; ^4^ College of Veterinary Medicine, Shanxi Agricultural University, Taigu, China; ^5^ Faculty of Medicine and Health Sciences, School of Veterinary Medicine and Science, University of Nottingham, Loughborough, United Kingdom; ^6^ Key Laboratory of Veterinary Public Health of Higher Education of Yunnan Province, College of Veterinary Medicine, Yunnan Agricultural University, Kunming, China

**Keywords:** *Fasciola gigantica*, peripheral blood mononuclear cells, RNA-seq, excretory-secretory products, long noncoding RNA

## Abstract

*Fasciola gigantica* produces excretory-secretory products (ESPs) with immune-modulating effects to promote its own survival. In this study, we performed RNA-seq to gain a comprehensive global understanding of changes in the expression of mRNAs, miRNAs, lncRNAs, and circRNAs in goat peripheral blood mononuclear cells (PBMCs) treated with *F. gigantica* ESPs. A total of 1,544 differently expressed mRNAs (790 upregulated and 754 downregulated genes), 30 differently expressed miRNAs (24 upregulated and 6 downregulated genes), 136 differently expressed circRNAs (83 upregulated and 53 downregulated genes), and 1,194 differently expressed lncRNAs (215 upregulated and 979 downregulated genes) were identified. Gene Ontology (GO) and Kyoto Encyclopedia of Genes and Genomes (KEGG) enrichment analyses revealed that *F. gigantica* ESPs altered the expression of genes associated with the host immune response, receptor signaling, disease and metabolism. Results from RNA-seq were validated by qRT-PCR. These findings provide an important resource for future investigation of the role of mRNAs and non-coding RNAs in mediating the immune-modulating effects of *F*. *gigantica* ESPs.

## Introduction

Fasciolosis, caused by *Fasciola gigantica* and *F. hepatica*, is a significant parasitic disease, causing huge economic losses to livestock and serious adverse impact on public health (1–3). Fasciolosis is prevalent in developing countries, particularly in Asia, Africa, Latin America, and the Caribbean (2, 4, 5). The annual global economic losses to livestock production caused by liver fluke infection exceed $3 billion (6). Despite the significant impact of fasciolosis, our knowledge of host defense mechanisms against *F. gigantica* infection remains limited. Exploring the molecular mechanisms of interaction between the host and liver flukes would enhance our understanding of the host immune response and assist in identifying potential targets for the development of therapeutic interventions against fasciolosis.

Liver flukes employ a raft of strategies to subvert the host’s immune response, including the secretion of excretory-secretory products (ESPs) to modulate the host’s immune responses, to facilitate their own survival and to establish long-term infection (7, 8). In a previous study, by using co-immunoprecipitation coupled with tandem mass spectrometry, we identified 14, 16, 9, and 9 proteins in *F. hepatica* ESPs (FhESPs) that can bind to cytokines IL2, IL17, IFN-γ, and goat peripheral blood mononuclear cells (PBMCs) (9). In addition, a proteomic analysis of FgESPs interacting with buffalo serum at different infection periods (42, 70, and 98 dpi) identified 18 proteins after *F. gigantica* infection, including 13 cathepsin proteins, 4 glutathione S-transferase proteins, and 1 calcium-binding protein (10).

To identify the role of these proteins in the long-term survival of *F. gigantica*, we cloned and expressed the recombinant *F. gigantica* cathepsin B (rFgCatB), 14-3-3 epsilon (rFg14-3-3e), Ras-related Rab10 (rFgRab10) and thioredoxin peroxidase (rFgTpx) proteins, and characterized their effects on various functions of the goat PBMCs (11–14). In the related liver fluke *F. hepatica*, tegumental antigen affects the maturation and antigen presentation to host dendritic cells (DCs) (15). Furthermore, *F. hepatica* fatty acid-binding protein inhibits the activation of Toll-like Receptor (TLR) 4 and suppresses the expression of inflammatory cytokines induced by lipopolysaccharide (LPS) (16).

Although ESPs play a significant role in supporting liver flukes’ evasion of the host immune response, the molecular mechanisms that are mediated by ESPs remain poorly defined. To reveal new aspects of the immuno-modulatory effects of *F. gigantica* ESPs, we investigated the expression profiles of messenger RNAs (mRNAs), microRNAs (miRNAs), circular RNAs (circRNAs), and long non-coding RNA (lncRNAs) in goat PBMCs following incubation with ESPs of *F. gigantica*. The results showed that ESPs can regulate the proliferation, immune response, and metabolism of treated PBMCs. Our data provide new information for further in-depth analysis of the role of ESPs in the immune-evasive strategy of *F. gigantica*.

## Materials and Methods

### Source and Culture Conditions of the PBMCs

Goat PBMCs were isolated as previously described (11). Briefly, healthy goat venous blood samples mixed with ethylene diamine tetraacetic acid (EDTA) were collected into vacutainer tubes. Goat PBMCs were isolated using a commercial kit (TBD, Tianjin, China) according to the manufacturer’s instructions. The isolated PBMCs were cultured in RPMI 1640 medium containing 10% fetal bovine serum (FBS) and 1% penicillin-streptomycin (Gibco, California, USA) and were incubated in a humidified atmosphere of 5% CO_2_ at 37°C for 4 h.

### Preparation of ESPs

ESPs of *F. gigantica* were prepared according to a previously described method (15, 17–19). Briefly, adult liver flukes of *F. gigantica* were obtained from the bile ducts of slaughtered buffaloes naturally infected by *F. gigantica* and washed three times with phosphate-buffered saline (PBS) at 37°C. The washed adult flukes were then transferred to PBSG buffer (PBS containing glucose, supplemented with 10,000 UI/ml penicillin G, 10 mg/ml streptomycin sulfate, and 25 μg/ml amphotericin B) and incubated in plastic petri dishes (1 worm/2 mL) for 2 h at 37°C. The supernatant was collected three times (i.e., every 2 h over a 6-h period). After incubation, the supernatant was collected and centrifuged at 10,000*g* for 30 min at 4°C, then filter-sterilized with a 20-μm nylon filter. After filter-sterilization, ESPs were concentrated using Amicon^®^ Ultra-15 centrifugal filter devices (30 K), and the concentration of ESPs was measured by the Bicinchoninic Acid Protein Assay (BCA-1, Sigma-Aldrich, Corp., St. Louis, MO, USA) and stored at −80°C until analysis.

### RNA Extraction and RNA-seq

PBMCs (1 × 10^6^) were incubated with 80 µg of *F. gigantica* ESPs or an equal volume of PBS (controls) at 37°C with 5% CO_2_ for 24 h. This experiment was performed in triplicate. Total RNA of goat PBMCs was extracted using the Ribo-Zero Kit (Ambion, Austin, Tex, USA) following the manufacturer’s protocol. RNA integrity was evaluated using the Agilent 2100 Bioanalyzer (Agilent Technologies, Santa Clara, CA, USA). Samples with a RNA Integrity Number (RIN) ≥ 7 were included in the analysis. The sequencing libraries were constructed using TruSeq Stranded Total RNA with Ribo-Zero Globin, according to the manufacturer’s instructions. These libraries were sequenced on an Illumina sequencing platform (HiSeq 2500).

### Data Processing and Differential Expression Analysis

Raw reads generated from high-throughput sequencing were fastq format sequences. In order to obtain high-quality reads, trimmomatic software was used to remove low-quality bases, N-bases, and low-quality reads (20). HISTA2 was used to align clean reads to the goat reference genome (21). Also, Q20, Q30, and GC-content of the raw data were calculated (22). The “estimateSizeFactors” function of the DESeq R package was used to normalize the read counts (23), and the “nbinomTest” function was used to calculate P values and fold change values. The transcript with P-values < 0.05 and |log_2_ Fold change| > 1 was deemed to be a differentially expressed (DE) transcript.

### miRNA Prediction

The small RNA tags were mapped to a reference sequence by Bowtie (24) to analyze their expression and distribution on the reference genome. The data of miRBase20.0 were used as miRNA reference, and modified software miRDeep2 (25) was used to identify the known miRNAs. The software miREvo (26) and miRDeep2 (25) were combined to predict novel miRNA by exploring the secondary structure, the Dicer cleavage site and the minimum free energy of the small RNA tags unannotated in the former steps.

### CircRNA Prediction

In order to generate a SAM file, BWA software was used to align the sequencing reads of each sample to the reference genome (27). Then, the CIRI software was used to scan paired chiastic clipping (PCC) signals, and circRNA sequences were predicted based on junction reads and GT-AG cleavage signals (28).

### lncRNA Prediction

Stringtie software was used to assemble the known and new transcripts (29). The candidate lncRNA transcripts were screened by comparing with the gene annotation information of the reference sequence produced by Cuffcompare software (30). The transcripts with coding potential were then screened out using CPC (31), CNCI, Pfam (32), and PLEK (27) in order to obtain lncRNA predicted sequences.

### GO and KEGG Enrichment Analysis

Gene Ontology (GO) enrichment was used for functional annotation analysis. GOseq based Wallenius non-central hyper-geometric distribution (33), which adjusts for gene length bias, was implemented for GO enrichment analysis. The Kyoto Encyclopedia of Genes and Genomes (KEGG) (34) database (http://www.genome.jp/kegg/) was utilized to test the statistical enrichment of the target genes in KEGG pathways using KOBAS software (35).

### Quantitative Real-Time PCR (qRT-PCR)

qRT-PCR was performed to validate the transcriptome data using the SYBR assay according to the manufacturer’s instructions. The qRT-PCR reactions were carried out using TB Green^®^ Premix Ex Taq™ II (Tli RNaseH Plus) (Takara, Shuzo, Kyoto, Japan) with the following reaction conditions: 95°C for 30 s, followed by 40 cycles at 95°C for 5 s and 60°C for 30 s. The *GADPH* gene was used for mRNA, circRNA, and lncRNA normalization, while the *U6* gene was used for miRNA’s normalization. Three independent replicates were examined for each gene and the relative expression of the target mRNAs, mircRNAs, circRNAs, and lncRNAs was calculated using the 2^−ΔΔCT^ method. All primers used in this study are listed in **Table 1**.

**Table 1:** qRT-PCR primers used in this study.

Target Gene	Forward primer (5′-3′)	Reverse primers (5′-3′)
HDAC1	CAGGATCCTTATCGTTGACTGG	CGGTGCCAGGAGAAGTAAAG
MCM2	TGGCCAAGAAGGACAACAA	AGCGATGCTGGCAAAGAT
CD47	CCTCCTACACCATCAGCATAC	GACCATGCACTGGGATACAT
GAPDH*	CACCACACCTTCTACAAC	TCTGGGTCATCTTCTCAC
chi-miR-16a-3p	CGCGCGCGAAATTATCTCCAGTATTA	
chi-miR-211	CCTTCCCTTTGTCATCCTTTGCCC	
chi-miR-155-3p	CTGGGTCTGGCTCCTACATGTT	
U6*	CAAGGATGACACGCAAATTCG	
novel_circ_0017223	CACCTGTGCCGAGAGCTGTA	CCTGGGCAGCTGGTTTCTGA
novel_circ_0007701	GAAGAAGCTGCCATGAGAAGAG	TGCTGAAGCTGCCGAATATC
novel_circ_0003459	AGATGCTCGCTGCTGCTCTC	ATGAGACGTACGTGTCCCGG
ENSCHIT00000005943	TCAGTGTGCGGTTGACTTAG	CAACCCTCTGCCTTCTCAAA
LNC_012500	CGTAACGCGAATCGCAAAC	TGAGGATCGGGTTAAGGGT
ENSCHIT00000002081	AGACTGTGTCCAGAGGTAACTA	GCTGTGAAAGATGGTGAGTAAATG

*The GADPH gene was used for mRNA, circRNA and lncRNA normalization, while the U6 gene was used for miRNA’s normalization.

### Cell Proliferation Assay by CCK-8 Kit

The effect of *F. gigantica* ESPs on goat PBMC’s proliferation was investigated using the CCK-8 kit (Beyotime, Haimen, Jiangsu, China) as previously described (13). Briefly, goat PBMCs were incubated with *F. gigantica* ESPs at concentrations of 5, 10, 20, 40, and 80 µg/ml. Forty-eight hours later, 10 µl of CCK-8 reagent were added to each cell culture and incubated for 4 h. The OD_450_ of each well was measured using a microplate reader (Bio-Rad, California, USA).

### Statistical Analysis

Student’s *t*-test was used to analyze the data from qRT-PCR experiments using GraphPad Prism 5 software (GraphPad Software, Inc., San Diego, CA, USA). All data are presented as means ± standard deviations (SDs).

## Results

### ESPs of *F. gigantica* Promoted the Proliferation of PBMCs

**Figure 1:**
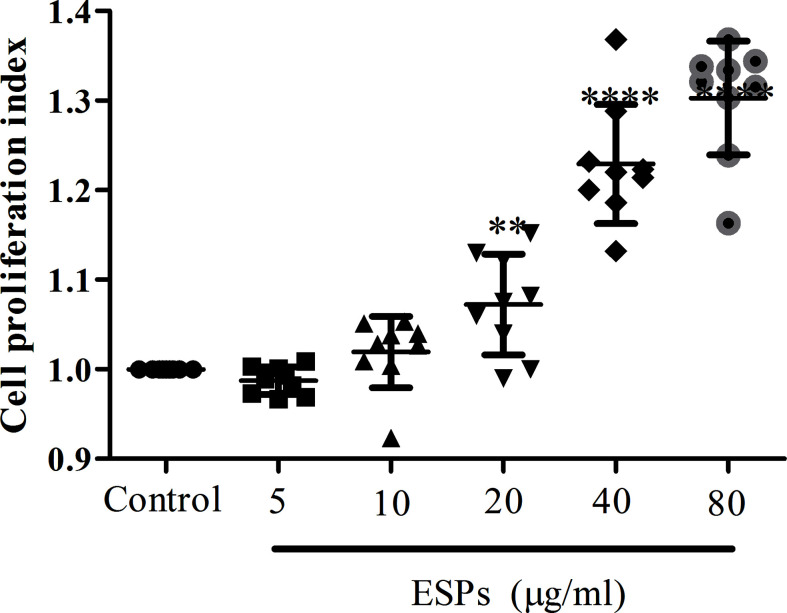
The ESPs of *F. gigantica* promote goat PBMC’s proliferation in a dose-dependent manner. PBMCs were treated with *F. gigantica* ESPs at the indicated range of concentrations for 48 h. Cell proliferation was quantified as described in the methods (*n* = 3). The error bars represent standard deviations. ** *p* < 0.01; **** *p* < 0.0001 compared to control untreated PBMCs.

*F. gigantica* ESPs promoted the proliferation of goat PBMCs in a concentration-dependent manner (**Figure 1**). Significant differences were detected between cells treated with 20 (*p* < 0.1), 40 (*p* < 0.0001) and 80 (*p* < 0.0001) µg/ml ESPs, when compared with untreated (control) cells. However, no significant differences were detected between cells exposed to lower concentrations (5 or 10 µg/ml) and control cells.

### DE mRNAs, miRNAs, circRNAs, and lncRNAs

**Figure 2:**
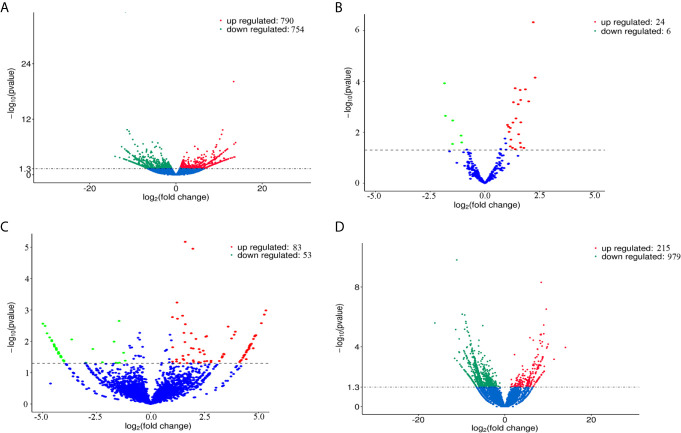
Volcano plots showing the differentially expressed mRNAs **(A)**, miRNAs **(B)**, circRNAs **(C)**, and lncRNAs **(D)** detected in goat PBMCs after treatment with *F. gigantica* ESPs. The negative log_10_ transformed P values (y-axis) are plotted against the average log_2_ fold changes in expression (x-axis). Data points representing RNAs that were not identified as differentially expressed are shown in blue. Transcripts that are differentially expressed after treatment with ESPs (corrected P value < 0.05) with an absolute log_2_ fold change (|FC|) greater than or less than 1 are shown as red (upregulated) and green (downregulated) dots.

**Figure 3:**
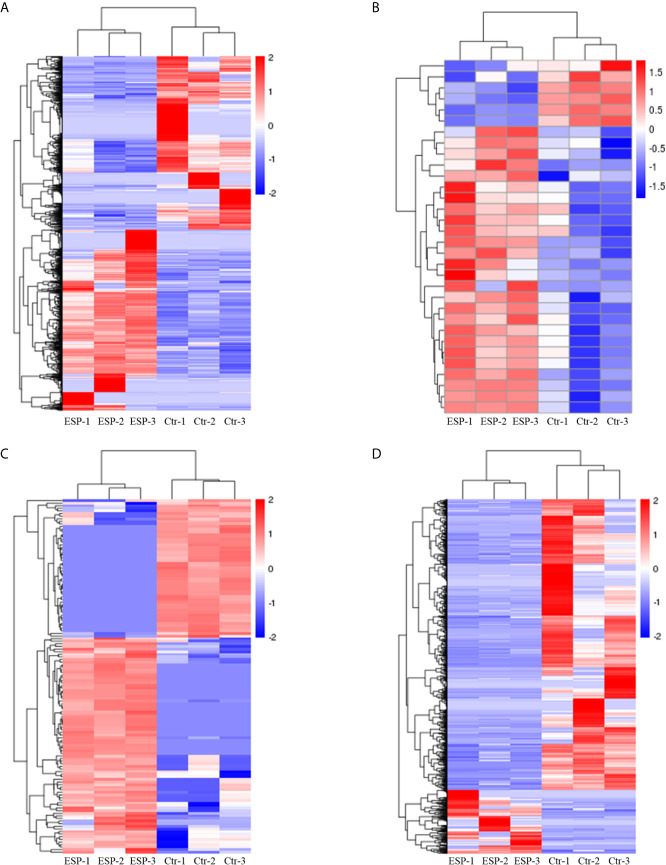
Hierarchical clustering heatmaps of the differentially expressed genes mRNAs **(A)**, miRNAs **(B)**, circRNAs **(C)**, and lncRNAs **(D)**. Each column represents the value for one separate experiment of the control and ESP-treated goat PBMCs. Each raw represents the individual RNAs. The colors represent the level of expression of the RNAs in the sample with red and blue denoting higher and lower expression levels, respectively.

The volcano plots (**Figure 2**) and heat maps (**Figure 3**) clearly showed the number and hierarchical clustering of differentiated mRNAs (**Figures 2A** and **3A**), miRNAs (**Figures 2B** and **3B**), circRNAs (**Figures 2C** and **3C**), and lncRNAs (**Figures 2D** and **3D**) in ESP-treated and control (untreated) groups. Our analysis identified 1,544 differently expressed mRNAs (including 790 upregulated and 754 downregulated, **Figure 2A** and **Supplementary Table S1**), 30 differently expressed miRNAs (including 24 upregulated and 6 downregulated, **Figure 2B** and **Supplementary Table S2**), 136 differently expressed circRNAs (including 83 upregulated and 53 downregulated, **Figure 2C** and **Supplementary Table S3**), and 1,194 differently expressed lncRNAs (including 215 upregulated and 979 downregulated, **Figure 2D** and **Supplementary Table S4**).

### Validation of DE mRNAs, miRNAs, circRNAs, and lncRNAs by qRT-PCR

**Figure 4:**
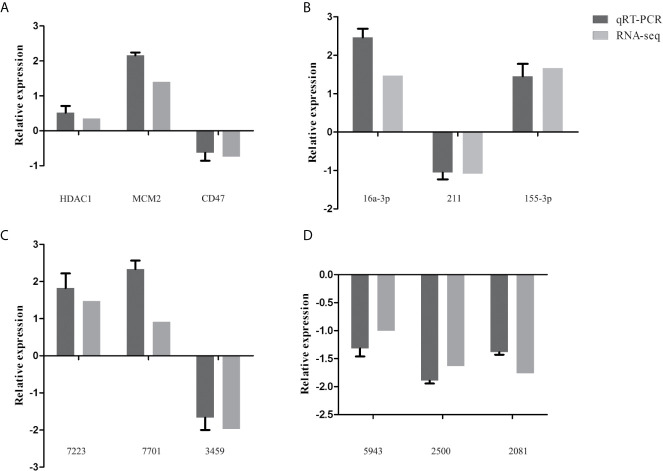
Validation of the expression of representative differentially expressed mRNAs (HDAC1, MCM2, CD47) **(A)**, miRNAs [16a-3p (chi-miR-16a-3p), 211 (chi-miR-211), 155-3p (chi-miR-155-3p)] **(B)**, circRNAs [7223 (novel_circ_0017223), 7701 (novel_circ_0007701), 3459 (novel_circ_0003459)] **(C)**, and lncRNAs [5943 (ENSCHIT00000005943), 2500 (LNC_012500), 2081 (ENSCHIT00000002081)] **(D)** using qRT-PCR. The x-axis shows the names of the selected RNAs and the y-axis shows the relative expression levels. The *GADPH* gene was used for mRNA, circRNAs, and lncRNA normalization, while the *U6* gene was used for miRNA’s normalization.

To validate the transcriptome data, a total of 12 genes were randomly selected for qRT-PCR verification, including three mRNAs (HDAC1, MCM2, CD47) (**Figure 4A**), three miRNAs [16a-3p (chi-miR-16a-3p), 211 (chi-miR-211), 155-3p (chi-miR-155-3p)] (**Figure 4B**), three circRNAs (7223 (novel_circ_0017223), 7701 (novel_circ_0007701), 3459 (novel_circ_0003459) (**Figure 4C**), and three lncRNAs [5943 (ENSCHIT00000005943), 2500 (LNC_012500), 2081 (ENSCHIT00000002081)] (**Figure 4D**). The qRT-PCR analysis showed that the trend of the data of qRT-PCR expression analysis was consistent with that of the data obtained by transcriptome analysis, supporting the accuracy of the transcriptomic data.

### GO and KEGG Analysis of the DE mRNAs

**Figure 5:**
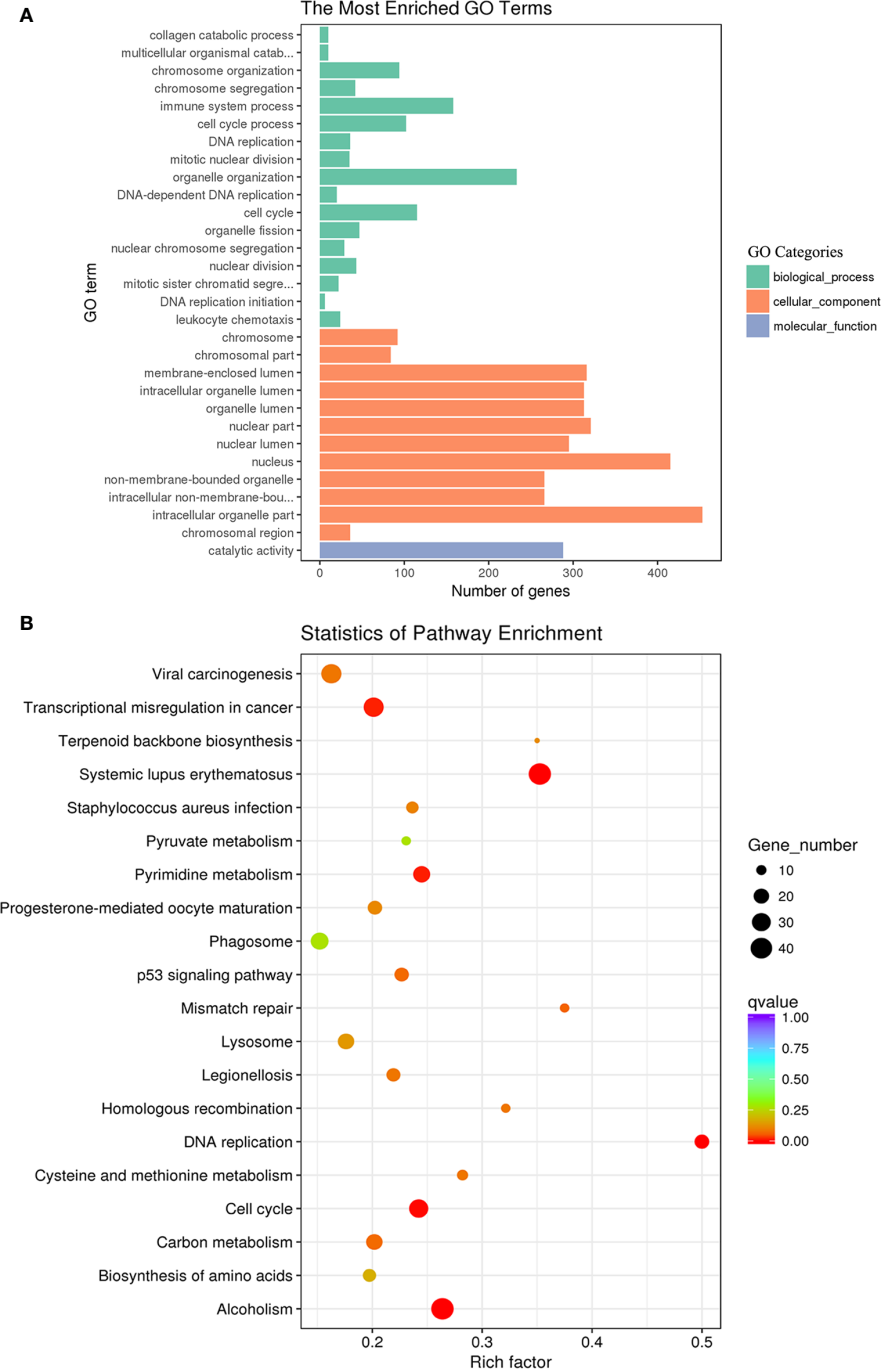
GO enrichment and KEGG pathway analyses of the differentially expressed mRNAs in goat PBMCs. **(A)** The 30 most enriched GO terms in the biological process, cellular component, and molecular function categories. **(B)** Scatterplot of KEGG pathway analysis of the top 20 pathways. The x-axis represents the pathway enrichment. The y-axis denotes the names of the significantly enriched pathways. The P-values are indicated by variations from blue to red. A deeper blue color indicates a greater significant difference.

To categorize and annotate the biological functions of the DE transcripts, GO enrichment analysis was performed. The GO terms were classed into three categories including biological process, cellular component and molecular function. The top 30 GO terms are shown in **Figure 5**. The DE mRNAs were significantly enriched in cellular component including the intracellular organelle part, nucleus and nuclear part (**Figure 5A**). Among these, the upregulated mRNAs were also significantly enriched in cellular component including the intracellular, intracellular part and organelles (**Supplementary Figure S1A**), and the downregulated mRNAs were significantly enriched in biological processes, such as the immune system process, regulation of localization, and regulation of the immune system process (**Supplementary Figure S1B**). The KEGG pathway enrichment was used to identify the significantly enriched pathways of the DE mRNAs. The top 20 pathways are shown in **Figure 5B**. Systemic lupus erythematosus, alcoholism, DNA replication and cell cycle were the most enriched pathways. Among them, the upregulated mRNAs were also significantly enriched in Systemic lupus erythematosus, alcoholism, DNA replication and cell cycle (**Supplementary Figure S1C**). The downregulated mRNAs were significantly enriched in *Staphylococcus aureus* infection, lysosome, complement and coagulation cascades, and phagosome (**Supplementary Figure S1D**).

### GO and KEGG Analysis of the Target Genes of miRNAs

**Figure 6:**
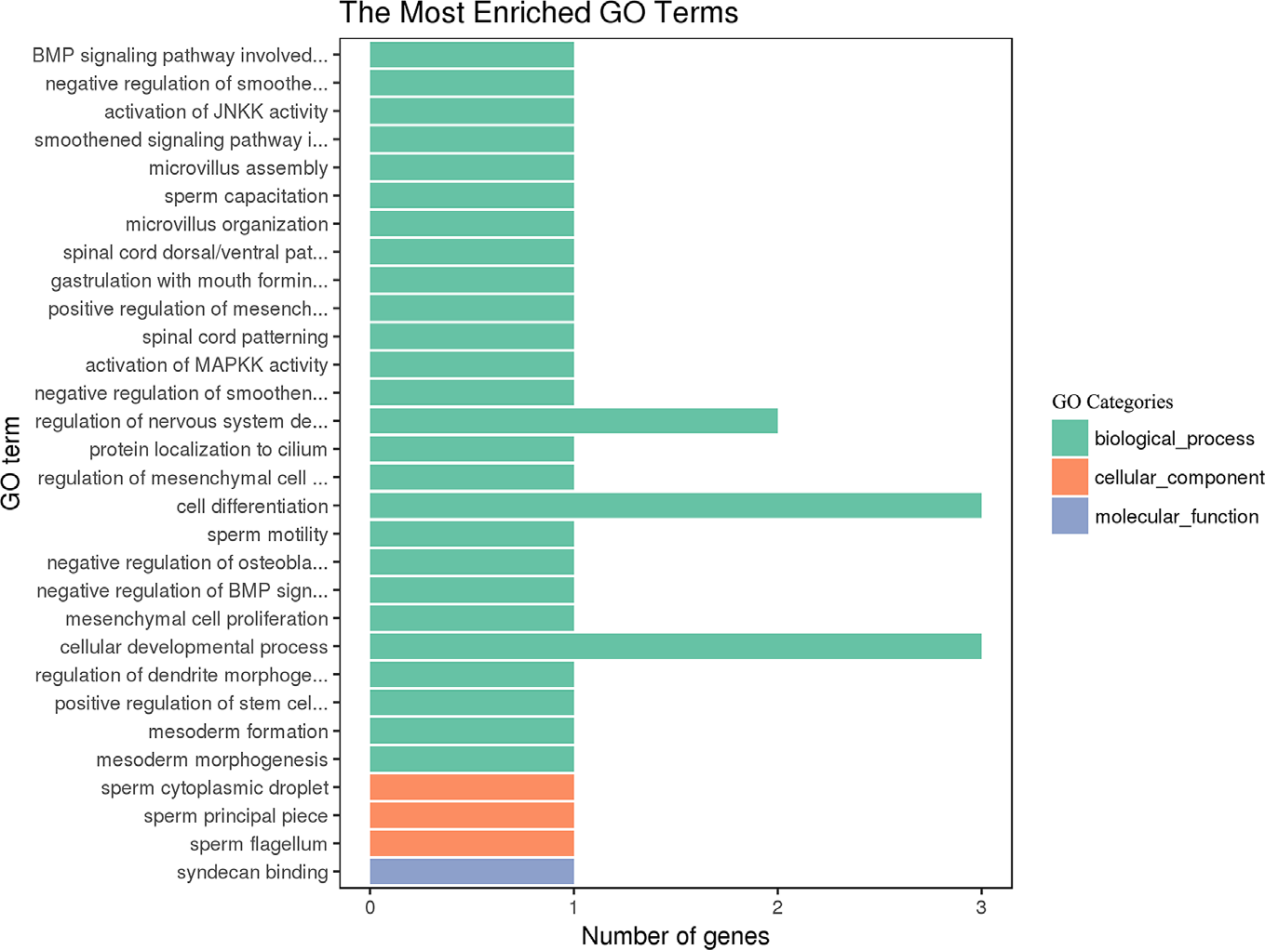
GO enrichment of the differentially expressed miRNAs in goat PBMCs. The 30 most enriched GO terms in the biological process, cellular component and molecular function categories. The y-axis represents the significantly enriched GO terms and the x-axis shows the number of genes in each GO term.

GO and KEGG pathway analysis was used to probe the main functions and the involved pathways of the DE miRNAs. As shown in **Figure 6**, the DE miRNAs were significantly enriched in biological processes, such as cell differentiation, cellular developmental process, and regulation of nervous system development. The most enriched pathway of the DE miRNA was the TGF-beta signaling pathway.

### GO and KEGG Analysis of the Target Genes of circRNAs

**Figure 7:**
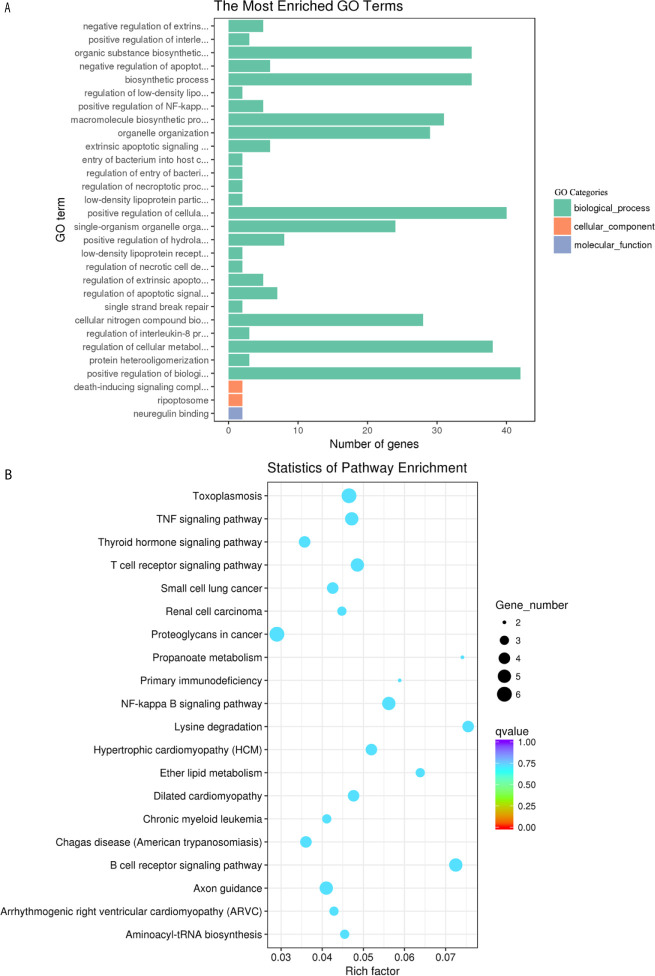
GO enrichment and KEGG pathway analyses of target genes of the differentially expressed circRNAs in goat PBMCs. **(A)** The 30 most enriched GO terms in the biological process, cellular component and molecular function categories of target genes of differentially expressed circRNAs. **(B)** Scatterplot of KEGG pathway analysis of the top 20 predominant pathways. The x-axis denotes the pathway enrichment. The y-axis represents the names of the significantly enriched pathways. The P-values are indicated by variations from blue to red. A deeper blue color indicates a greater significant difference.

The DE circRNAs were significantly enriched in biological processes, such as positive regulation of cellular process, positive regulation of biological process, and regulation of cellular metabolic process (**Figure 7A**). The most enriched pathways of the DE circRNAs involved included lysine degradation, the TNF signaling pathway, the B cell receptor signaling pathway, and hepatitis C (**Figure 7B**).

### GO and KEGG Analysis of the Target Genes of lncRNAs

**Figure 8:**
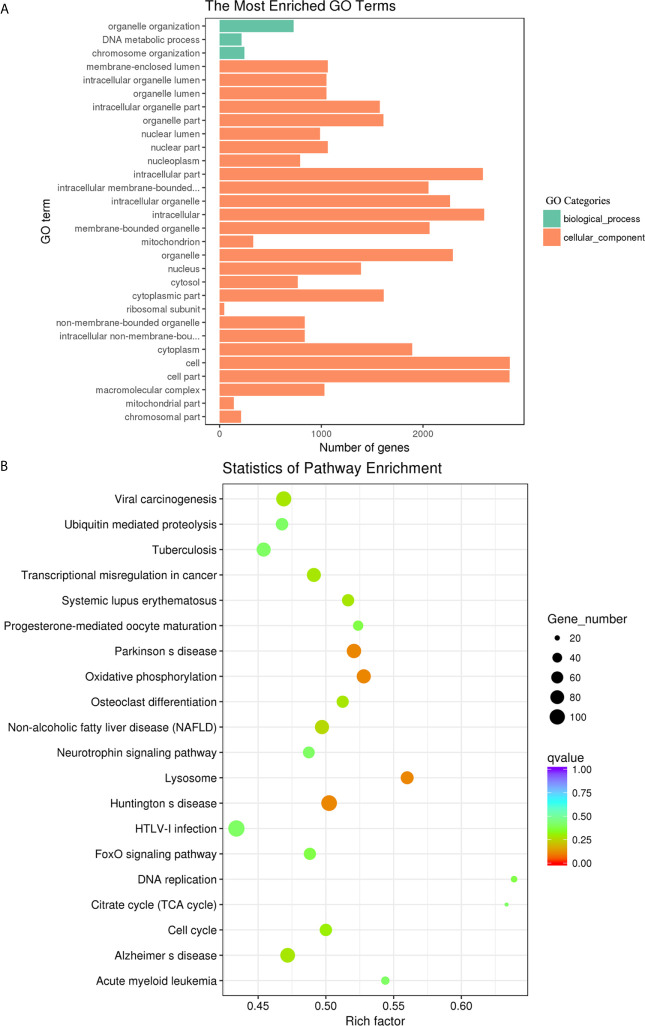
GO enrichment and KEGG pathway analyses of target genes of the differentially expressed lncRNAs in goat PBMCs. **(A)** The 30 most enriched GO terms in the biological process, cellular component and molecular function categories of target genes of differentially expressed lncRNAs. **(B)** Scatterplot of KEGG pathway analysis of the top 20 predominant pathways. The x-axis refers to pathway enrichment. The y-axis represents the names of the significantly enriched pathways. The P-values are expressed by variations from blue to red. A deeper blue color indicates a greater significant difference.

The biological functions of the DE lncRNAs were mainly involved in biological processes, such as negative regulation of biological process, organelle organization and macromolecular complex subunit organization (**Figure 8A**). Among them, the upregulated lncRNAs were also significantly enriched in biological process and cellular component, such as macromolecular complex subunit organization, chromosome and chromosome organization (**Supplementary Figure S2A**). The downregulated lncRNAs were significantly enriched in the categories biological process and molecular function, such as enzyme binding, kinase binding and single-organism cellular localization (**Supplementary Figure S2B**). The DE lncRNAs were significantly enriched in Type I diabetes mellitus, Influenza A and Allograft rejection (**Figure 8B**). Among them, the upregulated DE lncRNA were also significantly enriched in Influenza A, Type I diabetes mellitus and Graft-versus-host disease (**Supplementary Figure S2C**). The downregulated DE lncRNAs were significantly enriched in measles, rheumatoid arthritis, influenza A, and the chemokine signaling pathway (**Supplementary Figure S2D**).

## Discussion

In this study, we performed global RNA-seq to profile the expression of mRNAs, miRNAs, lncRNAs, and circRNAs in goat PBMCs exposed to *F. gigantica* ESPs. GO and KEGG enrichment analysis revealed that *F. gigantica* ESPs can influence the expression of transcripts associated with the host immune response, receptor signaling, cell proliferation, disease and metabolism. In previous studies, we have shown that the *F. gigantica* proteins, rFgRab10, rFgCatB and rFg14-3-34, influence cell proliferation and induce apoptosis in goat PBMCs (11–13). In the present study, we found that ESPs of *F. gigantica* promote cell proliferation as revealed by the CCK-8 assay (**Figure 1**) and upregulate cell proliferation-related genes (**Table 2**) of goat PBMCs. A previous study revealed that miR-211 suppresses epithelial ovarian cancer proliferation and cell-cycle progression by targeting Cyclin D1 and CDK6 (36), and miR-204-5p suppresses proliferation and invasion, and promotes chemotherapeutic sensitivity of colorectal cancer cells by downregulating RAB22A (37). The reduction in the expression of miR-211 and miR-204-5p in the present study implies a possible involvement of *F. gigantica* ESPs in promoting the proliferation of PBMCs.

**Table 2:** The relative expression of the differentially expressed genes in the corresponding key pathways.

Pathway name	Symbol	Description	Log_2_ (fold change)	P value
Cell Cycle	CDK2	Cyclin dependent kinase 2	1.264	0.0085
	CDC6	Cell division cycle 6	1.263	0.0151
	ORC6	Origin recognition complex subunit 6	1.314	0.0360
	ORC1	Origin recognition complex subunit 1	1.587	0.0455
	CDC25A	Cell division cycle 25A	1.887	0.0256
	CDC45	Cell division cycle 45	1.427	0.0185
	CHEK1	Checkpoint kinase 1	0.875	0.0296
	CHEK2	Checkpoint kinase 2	0.810	0.0033
	CCNA2	Cyclin A2	1.122	0.0026
	CCNB2	Cyclin B2	1.222	0.0086
	MCM2	Minichromosome maintenance complex component 2	1.402	0.0127
	BUB1B	BUB1 mitotic checkpoint serine/threonine kinase B	0.828	0.0001
	MCM7	Minichromosome maintenance complex component 7	1.144	0.0255
	CCNE1	Cyclin E1	1.228	0.0015
	ESPL1	Extra spindle pole bodies like 1, separase	1.612	0.0150
Toll-like receptor signaling pathway	IL12B	Interleukin 12B	2.791	0.0026
	TLR4	Toll like receptor 4	−1.478	0.0401
	TLR3	Toll like receptor 3	−1.737	0.0108
	IRF3	Interferon regulatory factor 3	−0.806	0.0470
	AKT1	AKT serine/threonine kinase 1	−0.805	0.0038
p53 signaling pathway	CCNB2	Cyclin B2	1.222	0.0086
	STRAP	Serine/threonine kinase receptor associated protein	0.248	0.0210
	CDK1	Cyclin dependent kinase 1	0.849	0.0056
	CHEK1	Checkpoint kinase 1	0.875	0.0296
	CHEK2	Checkpoint kinase 2	0.810	0.0033
	CCNE1	Cyclin E1	1.228	0.0015
	GADD45A	Growth arrest and DNA damage inducible alpha	1.675	0.0325
	GADD45B	Growth arrest and DNA damage inducible beta	0.745	0.0425
	CDK2	Cyclin dependent kinase 2	1.264	0.0085

TLR3 and TLR4 play a critical role in innate immunity and induction of inflammatory response (38). Previous studies have shown that parasite infections, such as *F. hepatica*, *F. gigantica*, *Leishmania*, and *Trypanosoma* (39–42), can modulate the expression of TLRs in order to evade the host’s innate immune response. For example, stimulation of human monocyte-derived DCs with live *Brugia malayi* microfilariae significantly downregulates mRNA expression of TLR3, TLR4, TLR5 and TLR7 (43). In cattle, *F. hepatica* ESP decreases TLR2/TLR4-mediated immune activation in order to limit excessive inflammation and attenuate the pathological reactions that accompany *F. hepatica* infection (44). Another study suggested that TLR4 is involved in *F. hepatica* fatty acid-binding protein (FhFABP) and induces alternative activation of human macrophages (45). FhFABP was suggested to suppress TLR4 activation in macrophages and inhibit the inflammatory cytokines induced following incubation with LPS (16). In the present study, PBMCs incubated with *F. gigantica* ESPs significantly downregulated mRNA expression of TLR3 and TLR4 (**Table 2**). TLR3 suppression promotes a Th2 immune response to *Schistosoma mansoni* infection. In *S. mansoni* infected TLR3 gene-deficient mice, Th1-associated cytokines (e.g., IL-12) are significantly decreased and Th2-associated cytokines and chemokines are significantly increased (46). rFgCatB protein (component of *F. gigantica* ESPs) can induce the expression of Th2-associated cytokines (e.g., IL-4, IL-10, and TGF-β) (13). These data suggest that reduced expression of TLR3 skewed the host immune response towards a Th2 response during *F. gigantica* infection.

Interferon regulatory factor 3 (IRF3) is an important transcription factor involved in the downstream regulation of My88-independent TRIF-dependent TLR signaling (46), which regulates the production of type I IFNs and the induction of inducible nitric oxide synthase (iNOS) expression (47, 48). iNOS catalyzes the conversion of arginine into citrulline and nitric oxide (NO), which protect the host against pathogen invasion (49). In this study, the expression of IRF3 was significantly decreased in PBMCs treated with *F. gigantica* ESPs. The reduced expression of IRF3 supports the link between inhibition of TRIF-dependent TLR3 signaling and decreased expression of NOS2 during *F. hepatica* infection (50, 51), which may be one mechanism employed by the liver fluke to avoid damage caused by host-generated NO. These findings suggest a possible mechanism for *F. gigantica* ESPs in suppressing TLR expression or its signaling cascade.

In this study, the expression of miR-148a-5p was upregulated. miR-148a can inhibit pro-inflammatory cytokines (52). Also, miR-148a-3p, together with miR-30a-5p, can repress pro-inflammatory NF-κB signaling and attenuate inflammation (53). Notably, in the present study, the NF-kB signaling pathway was suppressed. Therefore, the upregulation of miR-148a-5p detected in our study may suggest a role for *F. gigantica* ESPs in shifting Th1/Th2 towards the Th2 immune response.

Apoptosis of immune cells is an immunosuppressive strategy employed by *F. hepatica*, wherein the ESPs induce apoptosis in eosinophils *via* a caspase-dependent mechanism, resulting ROS-mediated mitochondrial-membrane potential loss (54). *F. gigantica* infection can induce apoptosis in sheep PMBCs *via* two distinct pathways: the extrinsic death receptor pathway, which is induced by the upregulation of TNF and TNFR1/TNFR2 (40, 50), and the intrinsic mitochondrial pathway (55). In our study, most death receptors and FAS receptor were downregulated, whereas the p53 signaling pathway-related genes were upregulated (**Table 2**), suggesting that *F. gigantica* ESPs may induce apoptosis through the p53 signaling pathway, rather than by the Fas/Fasl model. Further investigation of the role of p53 signaling pathway in *F. gigantica*–induced apoptosis of immune cells is warranted.

*Fasciola* infection can disrupt liver functions, resulting in disruption of the overall metabolism of infected livestock and losses in productivity (56). KEGG analysis revealed that exposure of goat PBMCs to *F. gigantica* ESPs upregulated genes related to lipid, nucleotide, amino acid, carbohydrate, and glycan metabolism. Alterations in metabolism during infection with *F. gigantica* and *F. hepatica* have been reported in previous studies (57–59). However, the mechanisms by which *F. gigantica* ESPs alter the host metabolic pathways remain to be elucidated.

Noncoding RNAs (miRNAs, circRNAs, and lncRNAs) participate in the regulation of different biological processes. In this study, a large number of circRNAs and lncRNAs were identified. However, the exact functions of these ncRNAs remain unclear. Pathways analysis indicated that the target genes of lncRNAs were highly enriched in disease-related pathways, such as toxoplasmosis, influenza A, and hepatitis B, and immune-related pathways, such as antigen processing and presentation, the Jak-STAT signaling pathway, and cytokine-cytokine receptor interaction. The target genes of circRNAs were highly enriched in the TNF signaling pathway and Hepatitis C. However, the target genes of miRNAs were only enriched in the TGF-beta signaling pathway. The immune-regulatory roles of these ncRNAs during *F. gigantica* infection need to be further investigated.

In conclusion, RNA-seq profiling of goat PBMCs treated with *F. gigantica* ESPs revealed significant changes in the expression of mRNAs, miRNAs, lncRNAs and circRNAs. The abovementioned *F. gigantica* ESP-induced changes appear to mediate a number of immune-regulatory mechanisms that enable *F. gigantica* to establish a persistent infection. These data should form the basis for further in-depth investigations of ESP-mediated immune-evasion strategies used by *F. gigantica* to subvert host immune responses.

## Data Availability Statement

The datasets presented in this study can be found in online repositories. The data is deposited in the NCBI repository, accession number is PRJNA686179.

## Ethics Statement

This study was reviewed and approved by the Animal Ethics Committee of Lanzhou Veterinary Research Institute, Chinese Academy of Agricultural Sciences (permit no. 2018-012).

## Author Contributions

X-QZ, DC, S-SW, and HE conceived and designed the experiments. S-SW and DC performed the experiments. J-JH, W-BZ, A-LT, and G-HZ contributed reagents/materials/analysis tools. S-SW and DC analyzed the data and wrote the paper. HE, J-JH, W-BZ, and X-QZ critically revised the manuscript. All authors read and approved the final version of the manuscript. All authors contributed to the article and approved the submitted version.

## Funding

Project financial support was provided by the National Key Basic Research Program (973 Program) of China (grant 2015CB150300), the Agricultural Science and Technology Innovation Program (ASTIP) (grant CAAS-ASTIP-2016-LVRI-03) and Yunnan Expert Workstation (grant 202005AF150041).

## Conflict of Interest

The authors declare that the research was conducted in the absence of any commercial or financial relationships that could be construed as a potential conflict of interest.

## Supplementary Material

Supplementary Table 1 List of all differentially expressed messenger RNAs (mRNAs).

Supplementary Table 2 List of all differentially expressed microRNAs (miRNAs).

Supplementary Table 3 List of all differentially expressed circular RNAs (circRNAs).

Supplementary Table 4 List of all differentially expressed long non-coding RNA (lncRNAs).

Supplementary Figure 1 GO enrichment and KEGG pathway analyses of differentially expressed mRNAs in goat PBMCs. The 30 most enriched GO terms of the upregulated **(A)** and downregulated **(B)** mRNAs stratified according to the biological process, cellular component and molecular function categories. Scatterplots of the KEGG pathway analysis of the top 20 predominant pathways of the upregulated **(C)** and downregulated **(D)** mRNAs. The x-axis denotes the pathway enrichment. The y-axis shows the names of the significantly enriched pathways. The P-values are indicated by variations from blue to red. A deeper blue color indicates a greater significant difference.

Supplementary Figure 2 GO enrichment and KEGG pathway analyses of target genes of differentially expressed lncRNAs in goat PBMCs. The 30 most enriched GO terms of the target genes of upregulated **(A)** and downregulated **(B)** lncRNAs in the biological process, cellular component and molecular function categories. Scatterplots of KEGG pathway analysis of the top 20 predominant pathways of the target genes of upregulated **(C)** and downregulated **(D)** lncRNAs. The x-axis shows the pathway enrichment. The y-axis represents the names of the significantly enriched pathways. The P-values are indicated by variations from blue to red. A deeper blue color indicates a greater significant difference.
