# Supplementary figures and images for: Fasciola gigantica–Derived Excretory-Secretory Products Alter the Expression of mRNAs, miRNAs, lncRNAs, and circRNAs Involved in the Immune Response and Metabolism in Goat Peripheral Blood Mononuclear Cells
Source: Front Immunol. 2021 Apr 12;12:653755. doi: 10.3389/fimmu.2021.653755 (PMC8072156; doi:10.3389/fimmu.2021.653755)

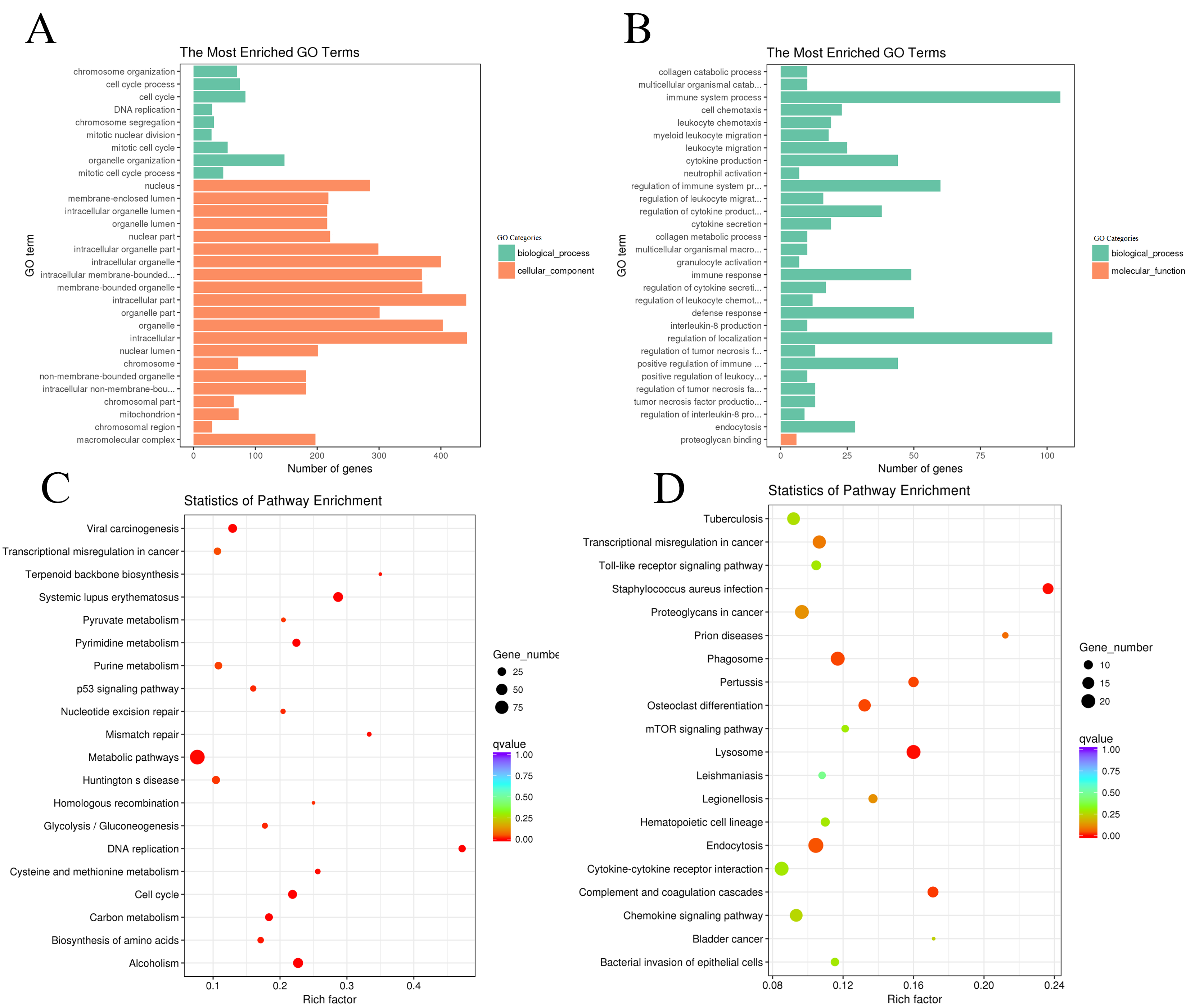

Supplement: Supplementary Figure 1 — GO enrichment and KEGG pathway analyses of differentially expressed mRNAs in goat PBMCs. The 30 most enriched GO terms of the upregulated (A) and downregulated (B) mRNAs stratified according to the biological process, cellular component and molecular function categories. Scatterplots of the KEGG pathway analysis of the top 20 predominant pathways of the upregulated (C) and downregulated (D) mRNAs. The x-axis denotes the pathway enrichment. The y-axis shows the names of the significantly enriched pathways. The P-values are indicated by variations from blue to red. A deeper blue color indicates a greater significant difference. [file Image_1.tif]

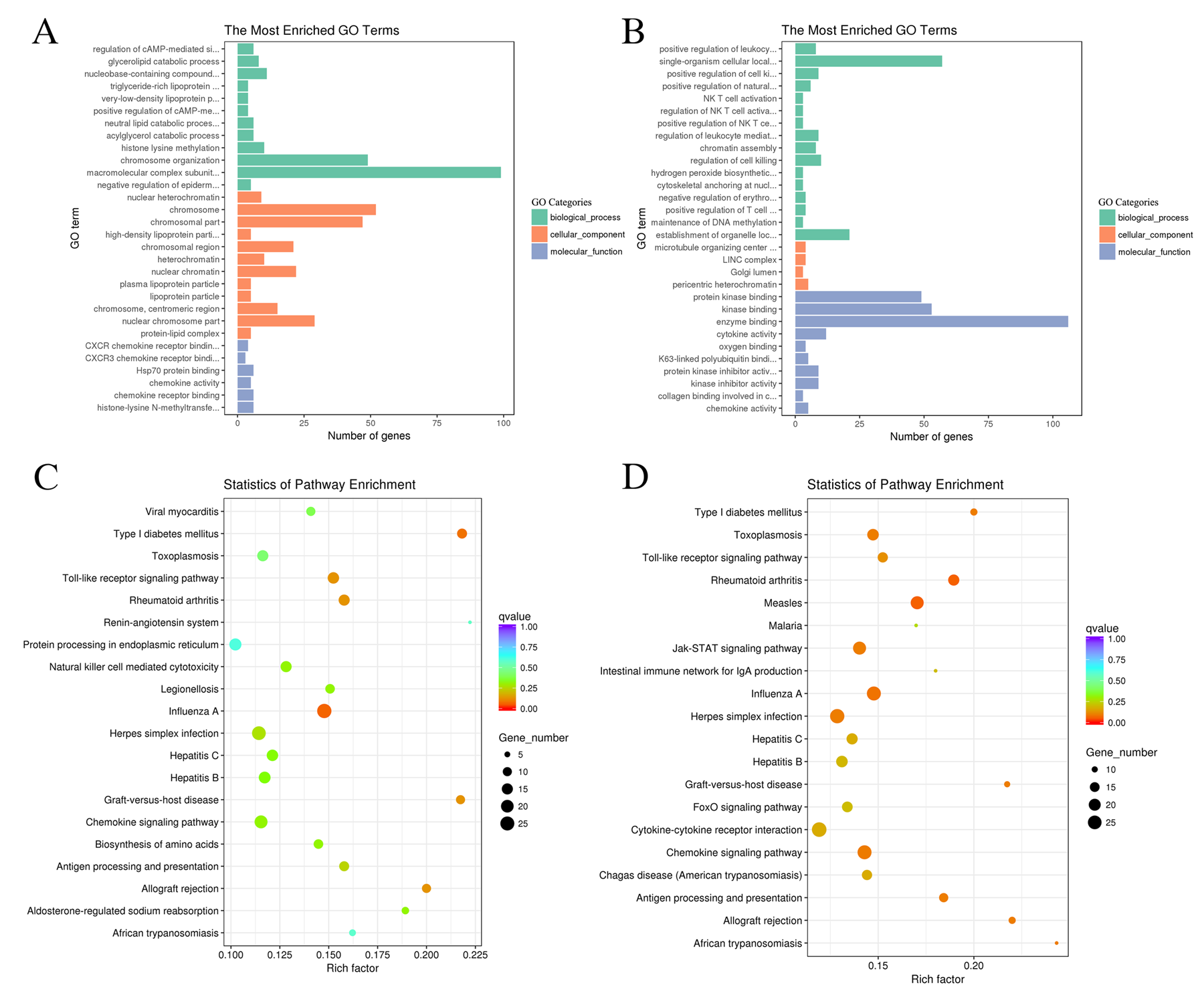

Supplement: Supplementary Figure 2 — GO enrichment and KEGG pathway analyses of target genes of differentially expressed lncRNAs in goat PBMCs. The 30 most enriched GO terms of the target genes of upregulated (A) and downregulated (B) lncRNAs in the biological process, cellular component and molecular function categories. Scatterplots of KEGG pathway analysis of the top 20 predominant pathways of the target genes of upregulated (C) and downregulated (D) lncRNAs. The x-axis shows the pathway enrichment. The y-axis represents the names of the significantly enriched pathways. The P-values are indicated by variations from blue to red. A deeper blue color indicates a greater significant difference. [file Image_2.tif]
